# The feasibility, reliability, and incremental value of two-dimensional speckle-tracking for the detection of significant coronary stenosis after treadmill stress echocardiography

**DOI:** 10.1186/s12947-021-00259-w

**Published:** 2021-07-23

**Authors:** Marc-André d’Entremont, Gabriel Fortin, Thao Huynh, Étienne Croteau, Paul Farand, Samuel Lemaire-Paquette, Marie-Claude Brochu, Doan Hoa Do, Serge Lepage, Warner Mbuila Mampuya, Étienne L. Couture, Michel Nguyen, Btissama Essadiqi

**Affiliations:** 1 Division of Cardiology, Department of Medicine, Sherbrooke University Hospital Center (CHUS) , 3001, 12e Avenue Nord, Sherbrooke, QC J1H 5N4 Canada; 2 Harvard T.H. Chan School of Public Health, Boston, MA USA; 3 McGill Health University Center, Montreal, QC Canada; 4 Sherbrooke University Hospital Research Center (CRCHUS), Sherbrooke, QC Canada

**Keywords:** Coronary artery disease, Exercise stress echocardiography, Speckle-tracking, Strain-imaging

## Abstract

**Background:**

Two-dimensional speckle-tracking echocardiography (STE) may help detect coronary artery disease (CAD) when combined with dobutamine stress echocardiography. However, few studies have explored STE with exercise stress echocardiography (ESE). We aimed to evaluate the feasibility, reliability, and incremental value of STE combined with treadmill ESE compared to treadmill ESE alone to detect CAD.

**Methods:**

We conducted a case–control study of all consecutive patients with abnormal ESE in 2018–2020 who subsequently underwent coronary angiography within a six-month interval. We 1:1 propensity score-matched these patients to those with a normal ESE. Two blinded operators generated a 17-segment bull's-eye map of longitudinal strain (LS). We utilized the mean differences between stress and baseline LS values in segments 13–17, segment 17, and segments 15–16 to create receiver operator curves for the overall examination, the left anterior descending artery (LAD), and the non-LAD territories, respectively.

**Results:**

We excluded 61 STEs from 201 (30.3%) eligible ESEs; 47 (23.4%) because of suboptimal image quality and 14 (7.0%) because of excessive heart rate variability precluding the calculation of a bull's-eye map. After matching, a total of 102 patients were included (51 patients in each group). In the group with abnormal ESE patients (mean age 66.4 years, 39.2% female), 64.7% had significant CAD (> 70% stenosis) at coronary angiogram. In the group with normal ESE patients (mean age 65.1 years, 35.3% female), 3.9% were diagnosed with a new significant coronary stenosis within one year. The intra-class correlation for global LS was 0.87 at rest and 0.92 at stress, and 0.84 at rest, and 0.89 at stress for the apical segments. The diagnostic accuracy of combining ESE and STE was superior to visual assessment alone for the overall examination (area under the curve (AUC) = 0.89 vs. 0.84, *p* = 0.025), the non-LAD territory (AUC = 0.83 vs. 0.70, *p* = 0.006), but not the LAD territory (AUC = 0.79 vs. 0.73, *p* = 0.11).

**Conclusions:**

Two-dimensional speckle-tracking combined with treadmill ESE is relatively feasible, reliable, and may provide incremental diagnostic value for the detection and localization of significant CAD.

**Supplementary Information:**

The online version contains supplementary material available at 10.1186/s12947-021-00259-w.

## Introduction

Exercise stress echocardiography (ESE) is a well-validated diagnostic method for detecting coronary artery disease (CAD) [1]. However, substantial expertise and experience are required to achieve optimal diagnostic accuracy [2]. Furthermore, a significant proportion of false-positive ESEs may occur, resulting in unnecessary normal coronary angiograms [3, 4]. The development of practical quantitative analysis techniques to increase the diagnostic yield of ESE has been a primary research focus [1]. Two-dimensional speckle-tracking echocardiography (STE), a quantitative measure of myocardial deformation, has been a recently developed method under investigation for the detection of CAD [5].

STE has not reliably demonstrated an increased accuracy compared to visual assessment for the detection of CAD with dobutamine stress echocardiography (DSE) [1, 6–8]. While researchers have explored the combination of STE with exercise stress echocardiography (ESE) with bicycle protocols, few have explored the combination with a treadmill protocol [9–11]. Furthermore, several studies that reported promising results of STE as a potential diagnostic technique have used unmatched controls [12–15]. As age, sex, diabetes, hypertension, smoking, and metabolic syndrome influence STE values, these studies may have been confounded by a higher burden of comorbidities in patients with CAD [16–20]. In addition, numerous studies have excluded patients with known CAD and baseline wall motion abnormalities (WMA), reducing the findings' potential generalizability [15, 21].

The majority of STE studies in DSE have used the global longitudinal strain (GLS) as a potential marker of ischemia [1]. However, the apical segments may be of greater interest because they typically display ischemic changes regardless of the location of the coronary stenosis [1]. Furthermore, a previous study suggested larger rest and stress variability in STE values in apical segments than mid-ventricular and basal segments, possibly increasing discrimination for significant coronary stenoses [22].

There is a need for studies that validate STE use in treadmill ESE. We aimed to conduct a pilot study to foremost evaluate the feasibility and interobserver reliability for STE in treadmill ESE. Secondly, as a hypothesis-generating analysis, we explored the incremental value of STE of the apical segments combined with the visual analysis of treadmill ESE compared to treadmill ESE alone to reclassify false-positive treadmill ESE.

## Material and methods

### Population and study design

We conducted a case–control study between March 2018 and March 2020. We included all consecutive patients with an abnormal ESE between March 2018 and March 2020 who subsequently underwent a coronary angiogram within a 6-month interval for the investigation of obstructive CAD at the Sherbrooke University Hospital Center (CHUS) in Quebec, Canada.

We included patients who had reached at least 85% of their maximum predicted heart rate with a normal ESE for the control group. Normal ESE controls were only selected from March 2018 to August 2018 to allow for a one-year ascertainment period. Therefore, patients with normal ESE from August 2018 to March 2020 were excluded. We ascertained new significant coronary artery stenoses by non-invasive testing (stress EKG, exercise stress echocardiogram, dobutamine stress echocardiogram or nuclear myocardial perfusion imaging) and coronary angiogram after the normal ESE using the hospital's electronic health records. Deaths were recorded as a competing risk. Controls who were diagnosed with a new coronary stenosis within one year despite a normal ESE were considered false negatives.

We only used the first ESE per patient for both groups and excluded all patients who underwent DSE, those who required contrast agent for ESE, and those with prior coronary artery bypass grafting (CABG). Of note, all patients with previously diagnosed CAD required a diagnosis by coronary angiogram and required subsequent complete percutaneous revascularization (all residual stenoses ≤ 50%) to allow for ascertainment of new significant coronary stenoses during follow-up. The study was approved by the Sherbrooke University Hospital Research Center (CRCHUS) ethics committee, and individual patient consent was not required.

### Treadmill exercise stress echocardiography

We performed ESE as per the recommendations of the American Heart Association (AHA) and the American Society of Echocardiography (ASE) guidelines [1, 23]. Images were obtained by five experienced sonographers using the GE Vivid E95 and M5Sc transducer (GE Vingmed Ultrasound AS, Horten, Norway) at baseline and within 90 s post-exercise with patients in the left lateral decubitus position. We recorded standard 2D grayscale images for the assessment of regional wall thickening in the apical four-chamber, apical two-chamber, apical long-axis, parasternal long- and short-axis views. We stored cine images of three representative cardiac cycles of the three apical views at baseline and post-exercise on an external hard drive for future analysis. The mean frame rate for all ESE was 48.7 ± 9 frames/sec.

Wall motion was evaluated at the time of examination by one of the six experienced echocardiographers in our laboratory. As the wall motion analysis was performed before angiography and STE, the echocardiographers were blinded to all other imaging results. Visual analysis was done at baseline and stress using all views and the 16-segment model as per guideline recommendations.^1^ We considered any stress-induced WMA as an abnormal result. In patients with baseline WMA, akinesis that became dyskinesis was not considered as an abnormal result as this generally corresponds to the mechanical response of an infarcted segment rather than ischemia [1]. Furthermore, patients with baseline WMA were considered as having a normal ESE if no additional WMA were observed during ESE.

### Two-dimensional speckle-tracking strain analysis

Two operators (MAD, GF), blinded to all other imaging results, performed 2D speckle-tracking on all eligible ESE using the automated function imaging (AFI) stress software version 201 (GE Vingmed Ultrasound AS, Horten, Norway) directly on the GE Vivid E95. The first operator (MAD) was a first-year general cardiology fellow who underwent formal echocardiography training and completed a ten-patient STE training set under the supervision of a senior echocardiographer (BE). The second operator (GF) was a final-year general cardiology fellow with level II certification. We manually traced the endocardial borders at the end-systolic frame in the three apical views at baseline and peak stress while optimizing the region of interest to better capture the myocardium. We defined the timing of aortic valve closure using the apical long-axis view. AFI software was then used to track the myocardial deformation and reject the poorly captured segments. We readjusted the endocardial tracing in the presence of poor tracking. We excluded all patients whose ESE had more than two rejected segments overall or any rejected apical segments at rest or stress. We also excluded all patients whose largest heart rate difference between two of the three apical images exceeded 30%, as this precluded the software from calculating the bull's-eye. Notably, the AFI software was unable to calculate the STE in the presence of a contrast agent.

**Fig. 1:**
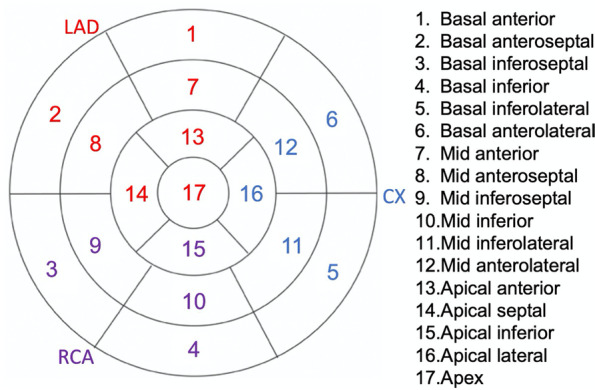
Schematic diagram of the 17 left ventricular segment model used in our study. LAD: Left anterior descending artery; CX: Circumflex artery; RCA: Right coronary artery

The software automatically calculated a 17-segment bull's-eye map and GLS. We defined segments 1, 2, 7, 8, and 13–17 as corresponding to the left anterior descending (LAD) artery, and segments 3, 4, 5, 6, and 9–12 as corresponding to the non-LAD territory using the standard template of coronary anatomy (Fig. 1) [1, 5]. Using the apical segments exclusively, we also defined segments 13–17 for the overall examination; segment 17 as corresponding to the LAD; and segments 15–16 as corresponding to the non-LAD territory. As we included patients with baseline WMA, we also calculated the delta strain values for standardization by subtracting the baseline values from the stress values. Stenosis of the left main coronary artery was considered to involve both the LAD and the circumflex coronaries.

### Coronary angiogram

An experienced interventional cardiologist interpreted the images from the diagnostic coronary angiogram. We defined significant CAD as ≥ 70% luminal diameter stenosis in at least one of the coronary arteries or as ≥ 50% in the left main coronary artery measured in the worst-view angiographic projection [24].

### Statistical analysis

To control for imbalances in baseline patient characteristics, we calculated propensity scores as the predicted probability of having an abnormal ESE using a multivariable logistic regression model. The covariates were chosen using a priori subject matter knowledge and included age, sex, hypercholesterolemia, hypertension, diabetes mellitus, current smoking status, and prior diagnosis of CAD. We matched 1:1 all patients with abnormal ESE to a normal ESE control using the optimal matching algorithm [25]. We assessed the covariate balance between the abnormal and normal ESE groups before and after propensity score matching using standardized differences [26].

We compared the clinical and ESE characteristics. Continuous data with normal distribution were presented as means with standard deviations and analyzed using the Student's t-test. Non-normal data were presented as medians with interquartile ranges and compared using the Wilcoxon rank-sum test. We performed chi-square tests for dichotomous variables unless the expected number of frequencies was less than 5, in which case we used a Fisher's exact test. We used a two-sided significance level of 0.05.

Interobserver variability in GLS and apical strain was determined by calculating the Pearson correlation coefficient, bias, limit of agreement, mean relative difference, and mean intra-class correlation coefficient. We calculated the mean intra-class correlation (ICC) using a two-way mixed-effect model measuring absolute agreement based on the mean rating [27].

We created receiver operating characteristic (ROC) curves to compare the diagnostic performance of STE and visual assessment. We used the mean between both operators for all strain measurements. Optimal cut-offs for sensitivity and specificity were determined using Youden's J statistic. For patients with abnormal ESE, the coronary angiogram was used as the gold standard. Patients with normal ESE were considered positive if they had a newly diagnosed significant coronary stenosis in the one-year ascertainment period, and negative otherwise.

To determine the incremental value of STE to reclassify the false-positive ESE, we considered a visually normal ESE to be negative. As a second step, we then used STE to further classify the abnormal ESEs as positive or not for significant CAD using the coronary angiograms as the gold standard. When combined with visual assessment, the optimal cut-offs of STE were only calculated for abnormal ESEs. We performed statistical testing to compare the areas under the curve (AUCs) based on the DeLong method and computed 95% confidence intervals [28]. We used a two-sided significance level of 0.05 and did not adjust for multiplicity. We conducted statistical analyses using SPSS version 26 (IBM Corporation, Armonk, NY, USA) and R version 4.0.2 (R Core Team, Vienna, Austria).

## Results

**Fig. 2:**
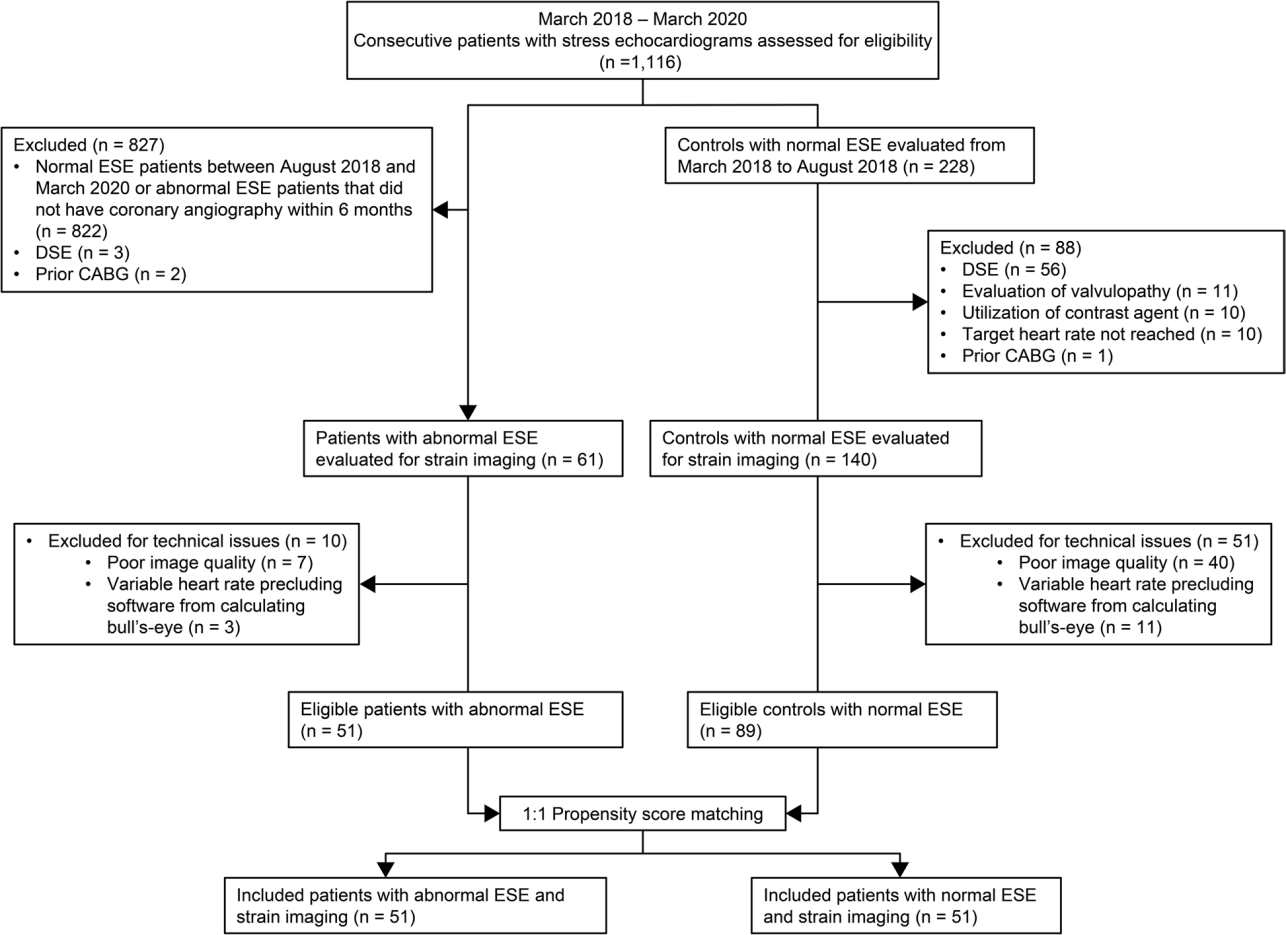
Study population flowchart. CABG: coronary artery bypass graft; DSE: dobutamine stress echocardiography; ESE: exercise stress echocardiography

We excluded 61 patients from the STE analysis from 201 (30.3%) eligible ESE due to technical issues; 47 (23.4%) because of suboptimal image quality and 14 (7.0%) because the heart rate was too variable between apical views precluding the software from calculating the bull's-eye (Fig. 2). Of note, STE analysis was possible for all patients at rest. Exclusions for suboptimal image quality was based on imaging at peak stress. After propensity score matching, there were 51 patients left in each group. Small differences (standardized differences ≥ 0.1) remained in terms of age, hypercholesterolemia, hypertension, and diabetes, but none were statistically significant (Table S1). The mean age of patients with abnormal ESE and normal ESE were 66.4 (39.2% female) and 65.1 years (35.3% female), respectively (Table 1).

**Table 1:** Clinical characteristics and rest echocardiographic parameters in the propensity score matched patients

Variables	Abnormal ESE (*n* = 51)	Normal ESE (*n* = 51)	*p*-values
**Clinical characteristics**			
Age (years) – mean (SD)	66.4 (8.7)	65.1 (9.4)	0.49
Female sex – no. (%)	20 (39.2)	18 (35.3)	0.84
Hypercholesterolemia – no. (%)	31 (60.8)	27 (53.0)	0.55
Hypertension – no. (%)	31 (60.8)	26 (51.0)	0.43
Diabetes mellitus – no. (%)	12 (23.5)	9 (17.7)	0.63
Current smoker – no. (%)	14 (27.5)	13 (25.5)	1.00
Prior hemodynamically significant CAD^a^ – no. (%)	13 (25.5)	14 (27.5)	1.00
**Medications at time of ESE**			
Beta-blocker – no. (%)	12 (23.5)	10 (19.6)	0.81
ACE inhibitor/ARB – no. (%)	25 (49.0)	18 (35.3)	0.23
Calcium channel blocker - no. (%)	15 (29.4)	6 (11.8)	0.05
Long-acting nitrate - no. (%)	3 (5.9)	3 (5.9)	1.00
**ESE parameters at rest**			
Left ventricular ejection fraction at rest – median (IQR)	55.0 (55.0–60.0)	55.0 (55.0–60.0)	0.77
Presence of ≥ 1 regional wall motion abnormalities at rest – no. (%)	15 (29.4%)	4 (7.8%)	0.005
Wall motion score index at rest – median (IQR)	1.0 (1.0 – 1.1)	1.0 (1.0 – 1.0)	0.008

*ACE* Angiotensin-converting enzyme, *ARB* angiotensin receptor blocker, *CAD* coronary artery disease, *ESE* exercise stress echocardiography, *SD* standard deviation

^a^ CAD required a diagnosis by coronary angiogram and subsequent complete revascularization by percutaneous coronary intervention

While both groups had similar baseline left ventricular ejection fractions and achieved comparable systolic blood pressures and target heart rates, the patients with positive ESE had more WMA at rest, attained lower metabolic equivalent (METS) levels and double product levels (Table 2). In the abnormal ESE group, 33 patients (64.7%) had significant CAD on angiography (Table 3). In the 1-year follow-up of the normal ESE group, 2 patients (3.9%) required revascularization. One patient had persisting unequivocal symptoms and underwent a positive nuclear myocardial perfusion imaging study with subsequent percutaneous revascularization, while the other had typical angina and underwent percutaneous revascularization directly. There were no deaths in the propensity score matched normal ESE group.

**Table 2:** Treadmill ESE parameters at peak stress

Variables	Abnormal stress echocardiogram (*n* = 51)	Normal stress echocardiogram CAD (*n* = 51)	*p*-values
**Stress EKG**			
Maximum heart rate achieved (bpm) – mean (SD)	141.9 (16.3)	148.5 (16.1)	0.04
Percentage of maximum predicted heart rate (%) – median (IQR)	92.0 (88.0 – 99.0)	94.0 (90.0 – 101.0)	0.08
Maximum systolic blood pressure (mmHg) – mean (SD)	169.4 (27.2)	174.4 (27.4)	0.36
Abnormal blood pressure response^a^ – no. (%)	7 (13.7)	3 (5.9)	0.18
Positive EKG for ischemia during the stress test – no. (%)	35 (68.6)	27 (52.9)	0.11
Clinically positive stress test^b^ – no. (%)	22 (43.1)	3 (5.9)	< 0.0001
Maximum achieved METS – median (IQR)	7.3 (6.1 – 9.6)	10.0 (7.7 – 11.7)	< 0.0001
Double product – mean (SD)	23,884.7 (4876.6)	26,179.9 (4705.6)	0.02
**Stress echocardiography**			
Absence of left ventricular ejection fraction increase at peak stress – no. (%)	31 (60.8)	0 (0.0)	< 0.0001
Wall motion score index at peak stress – median (IQR)	1.4 (1.2 – 1.5)	1.0 (1.0 – 1.0)	< 0.0001
Predicted significant CAD in the LAD territory – no. (%)	33 (64.7)	0 (0.0)	< 0.0001
Predicted significant CAD in the non-LAD territory – no. (%)	25 (49.0)	0 (0.0)	< 0.0001

*CAD* coronary artery disease, *EKG* electrocardiogram, *IQR* interquartile range, *METS* metabolic equivalents, *SD* standard deviation

^a^ Defined as the failure to reach a systolic blood pressure of 120 mmHg or to increase systolic blood pressure by more than 10 mmHg at peak stress or 3 min after the cessation of the stress test

^b^ Defined as angina or angina-like symptoms during stress treadmill stress test

**Table 3:** Coronary angiogram characteristics

**Variables**		**Overall**^**a**^ **(*n* = 53)**
Time between stress echocardiography and coronary angiogram (days) – median (IQR)		34.0 (14.0 – 63.0)
Coronary dominance	Right – no. (%)	44 (83.0)
	Left – no. (%)	5 (9.4)
	Co-dominance – no. (%)	4 (7.5)
Significant CAD overall – no. (%)		35 (66.0)
Significant CAD in the LAD territory – no. (%)		29 (54.7)
Significant CAD in the non-LAD territory – no. (%)		30 (56.6)
Significant CAD in the Cx territory – no. (%)		20 (37.7)
Significant CAD in RC the territory – no. (%)		20 (37.7)

*CAD* coronary artery disease, *Cx* circumflex artery, *IQR* interquartile range, *LAD* left anterior descending artery, *LM* left main artery, *RC* right coronary artery

^a^Patients who underwent coronary angiogram are comprised of the 51 patients with abnormal treadmill stress echocardiograms and two patients with normal treadmill stress echocardiograms

The interobserver reliability of STE is presented in Table 4. Overall, we demonstrated good-to-excellent interobserver reliability for the GLS as well as for the STE for the apical segments as demonstrated by the intra-class correlation coefficient. Bland–Altman plots are shown in the Supplementary material (Figures S1-S4).

**Table 4:** Interobserver variability for strain imaging

*r*	Bias	LOA	% mean relative difference	ICC (mean rating)
**Baseline GLS**				
0.79	0.63	± 3.81	-4%	0.87
**Baseline apical segments (segments 13–17)**				
0.74	1.15	± 6.74	-6%	0.84
**Peak stress GLS**				
0.85	0.09	± 4.12	-1.47%	0.92
**Peak stress apical segments (segments 13-17)**				
0.81	-0.78	± 9.24	4.19%	0.89

*ICC* intra-class correlation coefficient, *GLS* global longitudinal strain, *LOA* limit of agreement (± 1.96 standard deviations); % mean relative difference = (100 x (measure 1 – measure 2))/(measure 1)

**Fig. 3:**
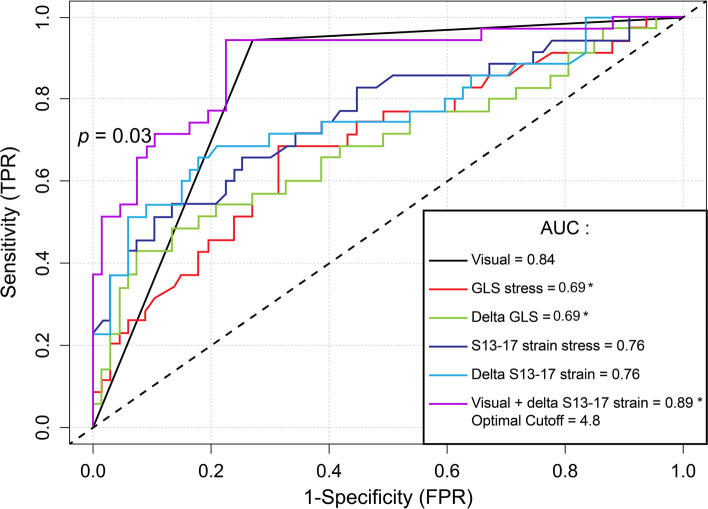
Receiver operator characteristic curve for the detection of significant coronary artery disease for the overall examination. *Denotes p < 0.05 compared to visual assessment

**Fig. 4:**
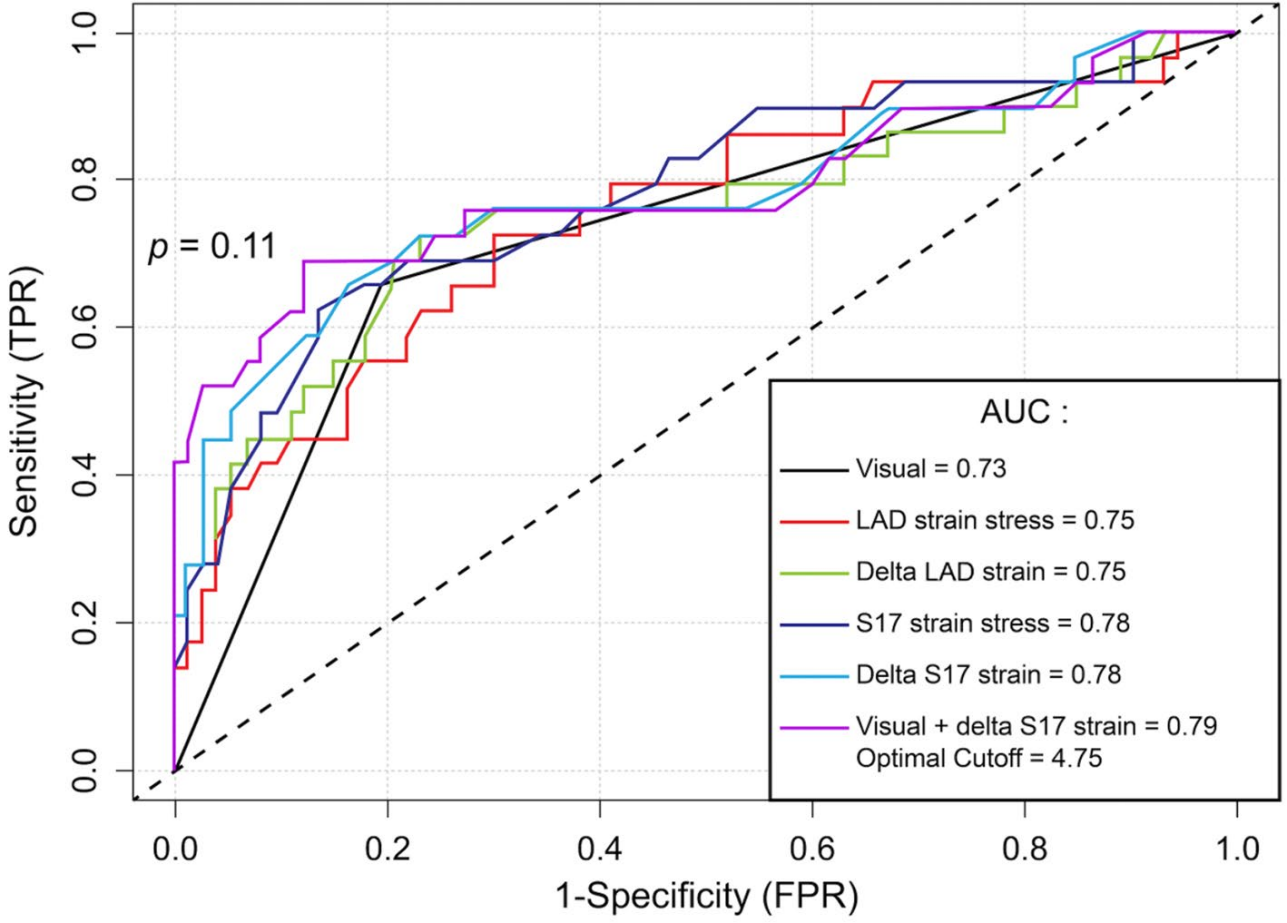
Receiver operator characteristic curve for the detection of significant coronary artery disease in the left anterior descending artery territory. *Denotes p < 0.05 compared to visual assessment

**Fig. 5:**
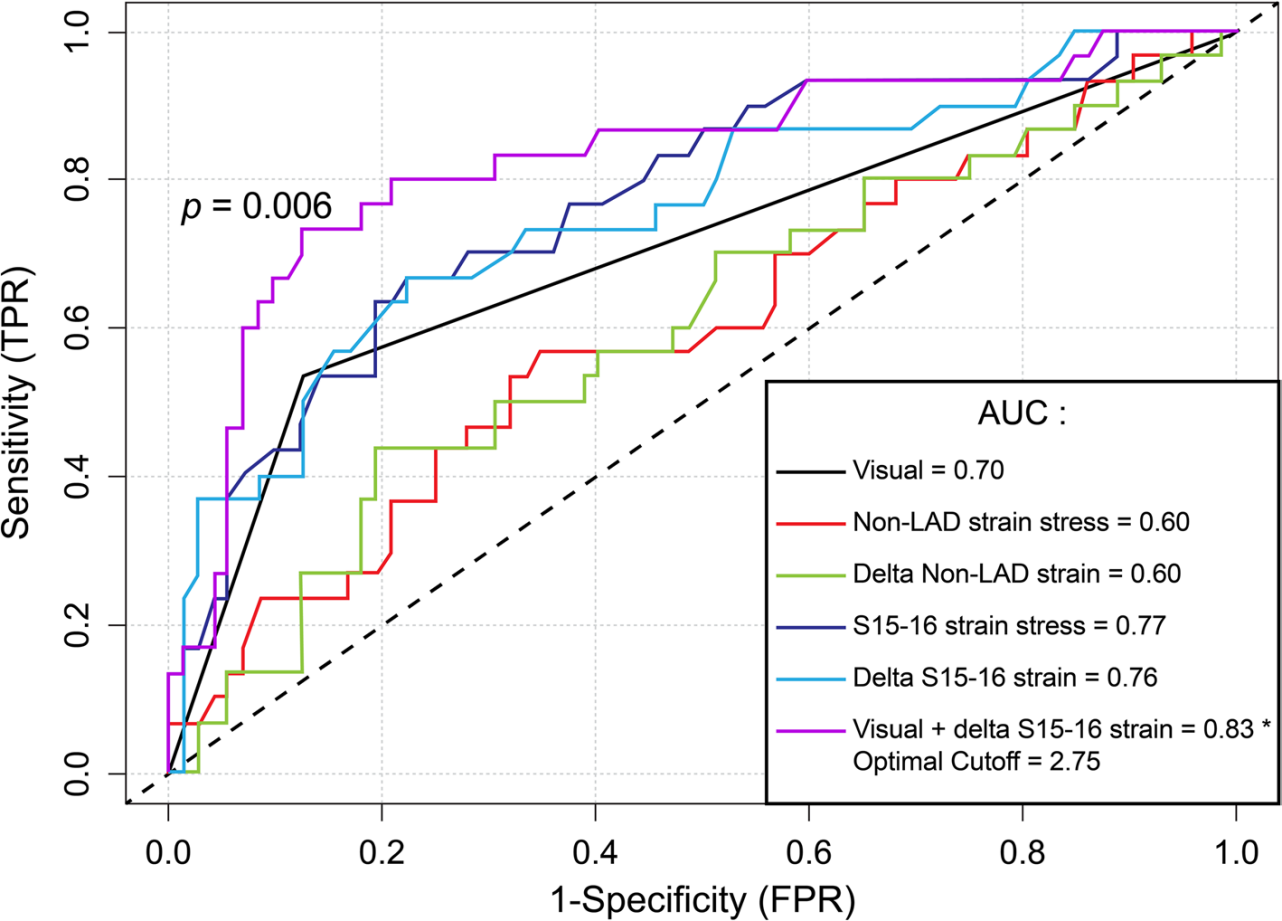
Receiver operator characteristic for the detection of significant coronary artery disease in the non-left anterior descending artery territory. *Denotes p < 0.05 compared to visual assessment

For the overall examination, the utilization of GLS alone at stress (AUC = 0.69 vs. 0.84, *p* = 0.006) and the delta GLS (AUC = 0.69 vs. 0.84, *p* = 0.01) resulted in inferior discrimination compared to visual assessment alone (Table 5). When considering normal ESE as negative and using STE to reclassify abnormal ESE, this combination was superior to visual assessment alone for the overall examination (AUC = 0.89 vs. 0.84, *p* = 0.025), for the non-LAD territory (AUC = 0.83 vs. 0.70, *p* = 0.006), but not for the LAD territory (AUC = 0.79 vs. 0.73, *p* = 0.11) (Figs. 3, 4 and 5). The sensitivity, specificity, negative predictive, and positive predictive values are shown in Table 5. Finally, we compared the strain characteristics of patients with significant CAD to those without significant CAD to demonstrate the increased variability and tendency toward larger differences between stress and rest values at the apex (Table S2).

**Table 5:** Diagnostic accuracy by visual and strain imaging for detecting significant coronary stenoses

	**Sensitivity** **(%)**	**Specificity** **(%)**	**PPV** **(%)**	**NPV** **(%)**	**Accuracy** **(%)**	**Optimal cut-off**	**AUC** **(95% CI)**	***p*-value**
**Visual assessment**								
Overall examination	94.3	73.1	64.7	96.1	80.4	N/A	0.84 (0.73 – 0.94)	Ref.
LAD	65.5	80.8	57.6	85.5	76.5	N/A	0.73 (0.66 – 0.80)	Ref.
Non-LAD	53.3	87.5	64.0	81.8	77.5	N/A	0.70 (0.66 – 0.75)	Ref.
**Stress GLS**								
Overall examination	68.6	68.7	53.3	80.7	68.7	-19.9	0.69 (0.58 – 0.80)	0.006
LAD	72.4	69.9	48.8	86.4	70.6	-21.2	0.75 (0.64 – 0.86)	0.63
Non-LAD	56.7	65.3	40.5	78.3	62.8	-15.0	0.60 (0.47 – 0.72)	0.09
**Delta GLS**								
Overall examination	42.9	92.5	75.0	75.6	75.5	4.1	0.69 (0.57 – 0.80)	0.01
LAD	72.4	76.7	55.3	87.5	75.5	1.1	0.75 (0.63 – 0.87)	0.67
Non-LAD	43.3	80.6	48.2	77.3	69.6	3.7	0.60 (0.47 – 0.72)	0.10
**Stress apical segment STE**								
Overall examination (S13-17)	51.4	89.6	72.0	77.9	76.5	-21.2	0.76 (0.65 – 0.86)	0.15
LAD (segment 17)	62.1	86.3	64.3	85.1	79.4	-21.8	0.78 (0.67 – 0.89)	0.23
Non-LAD (S15 and 16)	66.7	77.8	55.6	84.9	74.5	-22.8	0.77 (0.67 – 0.87)	0.38
**Delta apical segment STE**								
Overall examination (S13-17)	65.7	82.1	65.7	82.1	76.5	2.1	0.76 (0.65 – 0.86)	0.16
LAD (segment 17)	72.4	76.7	55.3	87.5	75.5	1.8	0.78 (0.66 – 0.89)	0.31
Non-LAD (S15 and 16)	66.7	77.8	55.6	84.9	74.5	1.9	0.76 (0.65 – 0.86)	0.49
**Visual assessment with delta apical segment strain STE if visual assessment positive**^**a**^								
Overall examination	94.3	77.6	68.8	96.3	83.3	4.8	0.89 (0.82 – 0.96)	0.025
LAD	69.0	87.7	69.0	87.7	82.4	4.8	0.79 (0.67 – 0.90)	0.11
Non-LAD	73.3	87.5	71.0	88.7	83.3	-2.8	0.83 (0.73 – 0.92)	0.006

*AUC* area under the curve, *GLS* global longitudinal strain, *PPV* positive predictive value, *NPV* negative predictive value, *S* segments, *STE* two-dimensional speckle-tracking echocardiography

^a^Cut-offs applicable only to echocardiograms judged to be positive by visual assessment

## Discussion

Our findings demonstrate that STE post-treadmill ESE is relatively feasible in patients who do not require a contrast agent, has good-to-excellent interobserver reliability between non-expert operators, and may provide reclassification value for patients with false-positive ESE. Furthermore, we showed that utilizing the STE difference between the apical segments at peak stress and baseline, in addition to visual assessment, maximized the diagnostic yield.

The sensitivity, specificity, and accuracy values of 94.3%, 73.1%, and 80.4% for the overall examination for visual assessment alone in our study are comparable to published estimates [1]. The high negative predictive value (96.1%) supports the excellent prognostic value for symptomatic obstructive CAD of a negative ESE [29]. However, the visual assessment sensitivity must be interpreted with caution because of the test verification bias and because the study sample was created by propensity score matching.

The combination of visual assessment and STE did lead to a slightly increased discrimination capacity. Compared to visual assessment alone, the combination of visual assessment and STE seemed to improve the accuracy of the detection of non-LAD stenoses in a clinically meaningful manner (77.5% vs. 83.3%). Of note, 30.3% of eligible patients who did not require a contrast agent were excluded, a higher proportion than typically displayed in DSE studies [1]. This significant exclusion of patients displayed the difficulty in obtaining high-quality images with little heart rate variability suitable for strain analysis in the ninety-second post-exercise window. However, our non-expert interobserver reliability was comparable to previous data of expert operators reflecting the intuitiveness of STE [30].

Our study is concordant with previous DSE studies, demonstrating that various strain measures may provide an incremental diagnostic yield to visual assessment [7, 31]. However, this has not been shown consistently by other authors [30]. Previous studies with promising results have utilized GLS [1, 32]. In our study, the use of peak stress GLS or the use of the difference between peak stress GLS and baseline GLS resulted in inferior discrimination than visual assessment for the overall examination. We hypothesize that our adjustment for confounding by selecting non-healthy controls may have attenuated the value of GLS for detecting significant CAD. Furthermore, a previous study that combined treadmill ESE and strain imaging did not utilize peak stress images and did not demonstrate conclusive results [10].

Our findings are in agreement with prior research that showed the utility of the apex (segment 17) for diagnosing LAD CAD disease, as well as the applicability of calculating the difference between peak and stress values in patients with WMA [6]. As ESE can provide important prognostic information by reproducing the physiological response with exercise, it is generally considered the test of choice over DSE [1]. Further studies are needed to validate the additional diagnostic value of strain imaging in treadmill ESE.

### Study limitations

The main limitation of our study was the absence of systematic coronary angiograms for all eligible patients. This was not feasible due to the study's retrospective nature. While we could not evaluate CAD in all patients with negative ESE, the one-year follow-up allowed us to examine for clinically driven diagnoses of coronary stenoses. For this reason, our diagnostic parameters must be interpreted with caution and viewed as hypothesis-generating. Our study may provide investigators with pilot data to conduct a prospective study with systematic angiograms to provide true estimates of speckle-tracking sensitivity in the post treadmill exercice context. Second, we could not complete STE in patients who required contrast and did not have optimal images, thus limiting our study's generalizability. Third, the visual assessment of the ESEs was only done by one single echocardiographer. Fourth, STE evaluations were performed by two non-expert operators. Finally, we did not achieve perfect correction of characteristics imbalance between the two groups of patients with standardized differences above 0.1 in some covariates. However, achieving standardized differences below 0.25 may be reasonable given our modest sample size [26].

## Conclusions

Two-dimensional speckle-tracking of the apical segments combined with treadmill ESE is relatively feasible in patients who do not require contrast, has good interobserver reliability, and may provide incremental diagnostic value for reclassifying false-positive ESE. Future prospective studies of ESE with two-dimensional speckle-tracking with larger sample sizes and coronary angiograms for all study participants are needed to confirm our findings.

## Supplementary Information

**Additional file 1: Table S1.** Clinical characteristics before and after propensity score matching **Table S2.** Strain characteristics of patients with significant CAD on coronary angiogram compared to patients without significant CAD on angiogram/patients with visually normal ESE. **Figure S1.** Bland-Altman plot for baseline global longitudinal strain. **Figure S2.** Bland-Altman plot for peak stress global longitudinal strain. **Figure S3.** Bland-Altmann plot for baseline strain of the apical segments (segments 13-17). **Figure S4.** Bland-Altmann plot for peak stress strain of the apical segments (segments 13-17).

## Data Availability

The data that support the study findings are available from the corresponding author upon reasonable request.

## Abbreviations

AUC: Area under the curve
CAD: Coronary artery disease
DSE: Dobutamine stress echocardiography
ESE: Exercise stress echocardiography
GLS: Global longitudinal strain
LAD: Left anterior descending artery
LS: Longitudinal strain
STE: Two-dimensional speckle-tracking echocardiography
ROC: Receiver operating characteristic
WMA: Wall motion abnormality
