# Supplementary material for: The feasibility, reliability, and incremental value of two-dimensional speckle-tracking for the detection of significant coronary stenosis after treadmill stress echocardiography
Source: Cardiovasc Ultrasound. 2021 Jul 23;19:27. doi: 10.1186/s12947-021-00259-w (PMC8305986; doi:10.1186/s12947-021-00259-w)
Supplement: Supplementary file 1 — Additional file 1: Table S1. Clinical characteristics before and after propensity score matching Table S2. Strain characteristics of patients with significant CAD on coronary angiogram compared to patients without significant CAD on angiogram/patients with visually normal ESE. Figure S1. Bland-Altman plot for baseline global longitudinal strain. Figure S2. Bland-Altman plot for peak stress global longitudinal strain. Figure S3. Bland-Altmann plot for baseline strain of the apical segments (segments 13-17). Figure S4. Bland-Altmann plot for peak stress strain of the apical segments (segments 13-17). [file 12947_2021_259_MOESM1_ESM.docx]

**Supplementary Material**

**The Feasibility, Reliability, and Incremental Value of Two-Dimensional Speckle-Tracking for the Detection of Significant Coronary Stenosis after Treadmill Stress Echocardiography**

**Table S1. Clinical characteristics before and after propensity score matching**

| **Variables** | **Unmatched**  **abnormal ESE**  **(n = 51)** | **Unmatched**  **normal ESE**  **(n = 89)** | **Standardized differences before matching** | **Matched**  **abnormal ESE**  **(n = 51)** | **Matched normal ESE**  **(n = 51)** | **Standardized differences after matching** | ***p*-values after matching** |
| --- | --- | --- | --- | --- | --- | --- | --- |
| Age (years) – mean (SD) | 66.4 (8.7) | 61.94 (11) | 0.51 | 66.4 (8.7) | 65.1 (9.4) | 0.14 | 0.49 |
| Female sex – no. (%) | 20 (39.2) | 39 (43.8) | 0.09 | 20 (39.2) | 18 (35.3) | 0.08 | 0.84 |
| Hypercholesterolemia – no. (%) | 31 (60.8) | 37 (41.6) | 0.39 | 31 (60.8) | 27 (53.0) | 0.16 | 0.55 |
| Hypertension – no. (%) | 31 (60.8) | 40 (44.9) | 0.32 | 31 (60.8) | 26 (51.0) | 0.20 | 0.43 |
| Diabetes mellitus – no. (%) | 12 (23.5) | 10 (11.2) | 0.29 | 12 (23.5) | 9 (17.7) | 0.14 | 0.63 |
| Current smoker – no. (%) | 14 (27.5) | 34 (38.2) | 0.24 | 14 (27.5) | 13 (25.5) | 0.04 | 1.00 |
| Prior CAD – no. (%) | 13 (25.5) | 19 (21.4) | 0.10 | 13 (25.5) | 14 (27.5) | 0.05 | 1.00 |

CAD, coronary artery disease; ESE, exercise stress echocardiography; SD. standard deviation.

**Table S2: Strain characteristics of patients with significant CAD on coronary angiogram compared to patients without significant CAD on angiogram/patients with visually normal ESE**

|  | **Overall** | **Significant CAD** | **No significant CAD or normal ESE** | ***p*-values** |
| --- | --- | --- | --- | --- |
| **GLS** | **(n = 102)** | **(n = 35)** | **(n = 67)** |  |
| STE at rest – median (IQR) | -19.8 (-21.8, -17.4) | -19.9 (-21.5, -17.3) | -19.8 (-21.9, -17.6) | 0.65 |
| Median STE at stress – median (IQR) | -20.2 (-22.2, -17.0) | -18.6 (-21.0, -15.3) | -21.0 (-22.5, -18.6) | 0.002 |
| Median difference stress/rest– median (IQR) | 0.6 (-1.8, 3.5) | 3.2 (-0.1, 5.8) | 0.0 (-2.0, 2.4) | 0.002 |
| **Segments 13-17** |  |  |  |  |
| STE at rest – median (IQR) | -25.3 (-27.9, -21.5) | -24.0 (-28.6, -21.3) | -25.4 (-27.7, -21.9) | 0.56 |
| Median STE at stress – median (IQR) | -26.2 (-29.6, -21.2) | -21.1 (-26.6, -15.5) | -27.8 (-31.1, -23.3) | <0.0001 |
| Median difference stress/rest– median (IQR) | -1.0 (-4.7, 4.3) | 5 (-2.9, 7.5) | -2.4 (-5.1, 1.2) | <0.0001 |
| **LAD territory** | **(n = 102)** | **(n = 29)** | **(n = 73)** |  |
| Median STE at rest – median (IQR) | -22.1 (-24.1, -18.7) | -22.0 (-23.8, -18.8) | -22.1 (-24.1, -18.7) | 0.91 |
| Median STE at stress – median (IQR) | -22.4 (-25.1, -18.5) | -18.8 (-22.3, -14.7) | -23.2 (-25.7, -19.9) | <.0001 |
| Median difference stress/rest – median (IQR) | -0.3 (-3.8, 3.3) | 3.9 (-0.4, 7.5) | -1.2 (-4.0, 1) | <0.0001 |
| **Segment 17** |  |  |  |  |
| Median STE at rest – median (IQR) | -25.0 (-28.0, -21.4) | -24.0 (-29.0, -21.3) | -25.5 (-28.0, -21.5) | 0.58 |
| Median STE at stress – median (IQR) | -26.3 (-29.6, -21.0) | -21.0 (-26, -15.0) | -28.0 (-31, -23.5) | <.0001 |
| Median difference stress/rest – median (IQR) | -1.0 (-4.5, 4.5) | 5.0 (-1.0, 11.0) | -2.5 (-5.0, 1.5) | <0.0001 |
| **Non-LAD territory** | **(n = 102)** | **(n = 30)** | **(n = 72)** |  |
| Median STE at rest – median (IQR) | -16.9 (-18.6, -15.4) | -16.5 (-18.3, -15.0) | -17.1 (-18.9, -15.6) | 0.50 |
| Median STE at stress – median (IQR) | -15.8 (-18.5, -13.3) | -14.6 (-17.6, -11.8) | -15.9 (-18.6, -13.7) | 0.13 |
| Median difference stress/rest – median (IQR) | 1.4 (-1.7, 3.7) | 2.3 (-0.9, 5.5) | 1 (-1.9, 3.3) | 0.12 |
| **Segments 15-16** |  |  |  |  |
| Median STE at rest – median (IQR) | -24.9 (-27.5, -21.6) | -23.5 (-27.6, -21.0) | -25.0 (-27.4, -22.1) | 0.52 |
| Median STE at stress – median (IQR) | -25.4 (-29.0. -21.0) | -21 (-25.2, -16.4) | -26.9 (-29.8, -23.3) | <0.0001 |
| Median difference stress/rest – median (IQR) | -0.6 (-4.0, 3.8) | 3.9 (-1.9, 9.1) | -2.13 (-4.4, 1.7) | <0.0001 |

CAD, coronary artery disease; IQR, Interquartile range; GLS, global longitudinal strain; LAD, left anterior descending artery; STE, two-dimensional speckle-tracking echocardiography

**Figure S1.** Bland-Altman plot for baseline global longitudinal strain

**
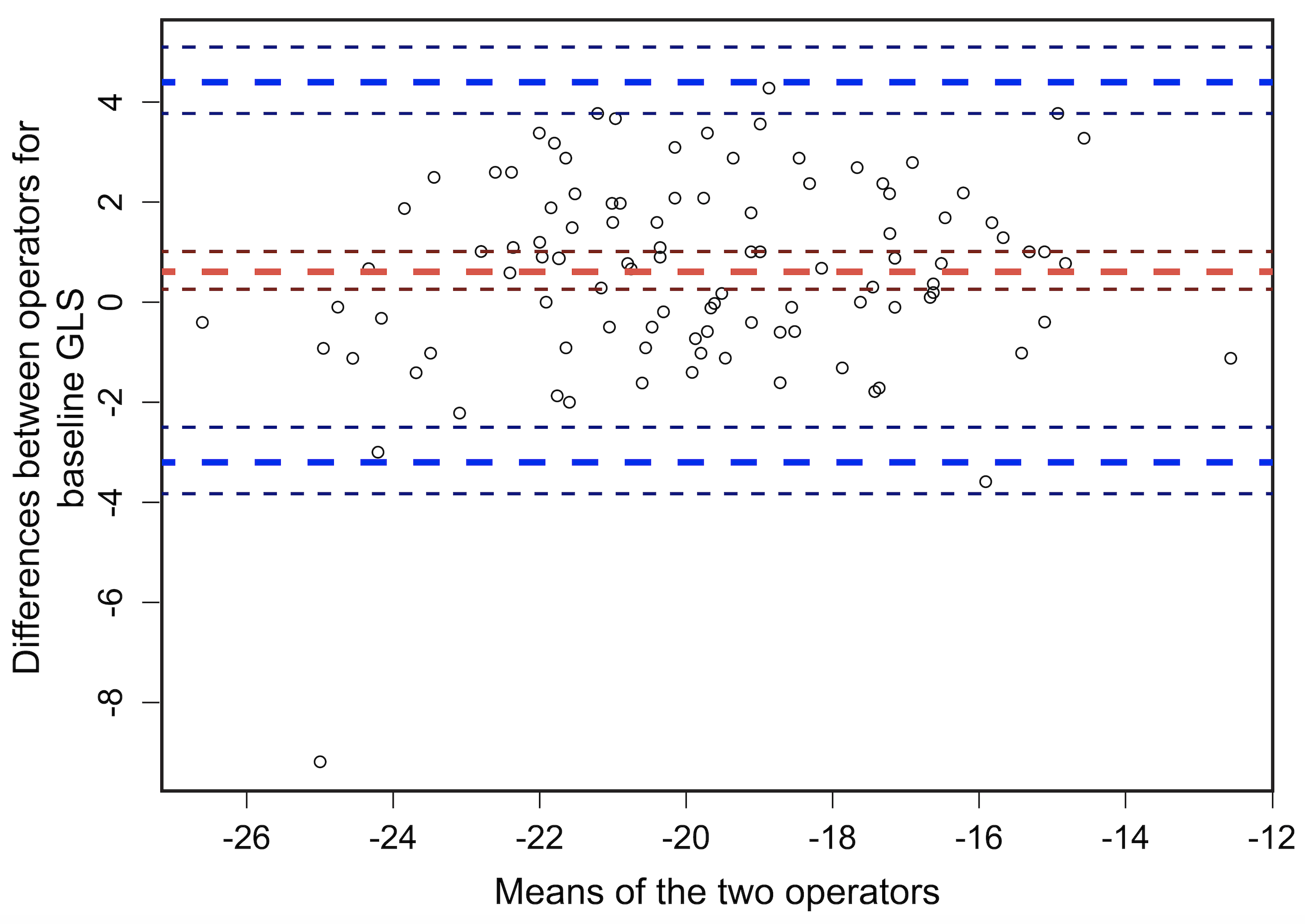
**

**Figure S2.** Bland-Altman plot for peak stress global longitudinal strain

**
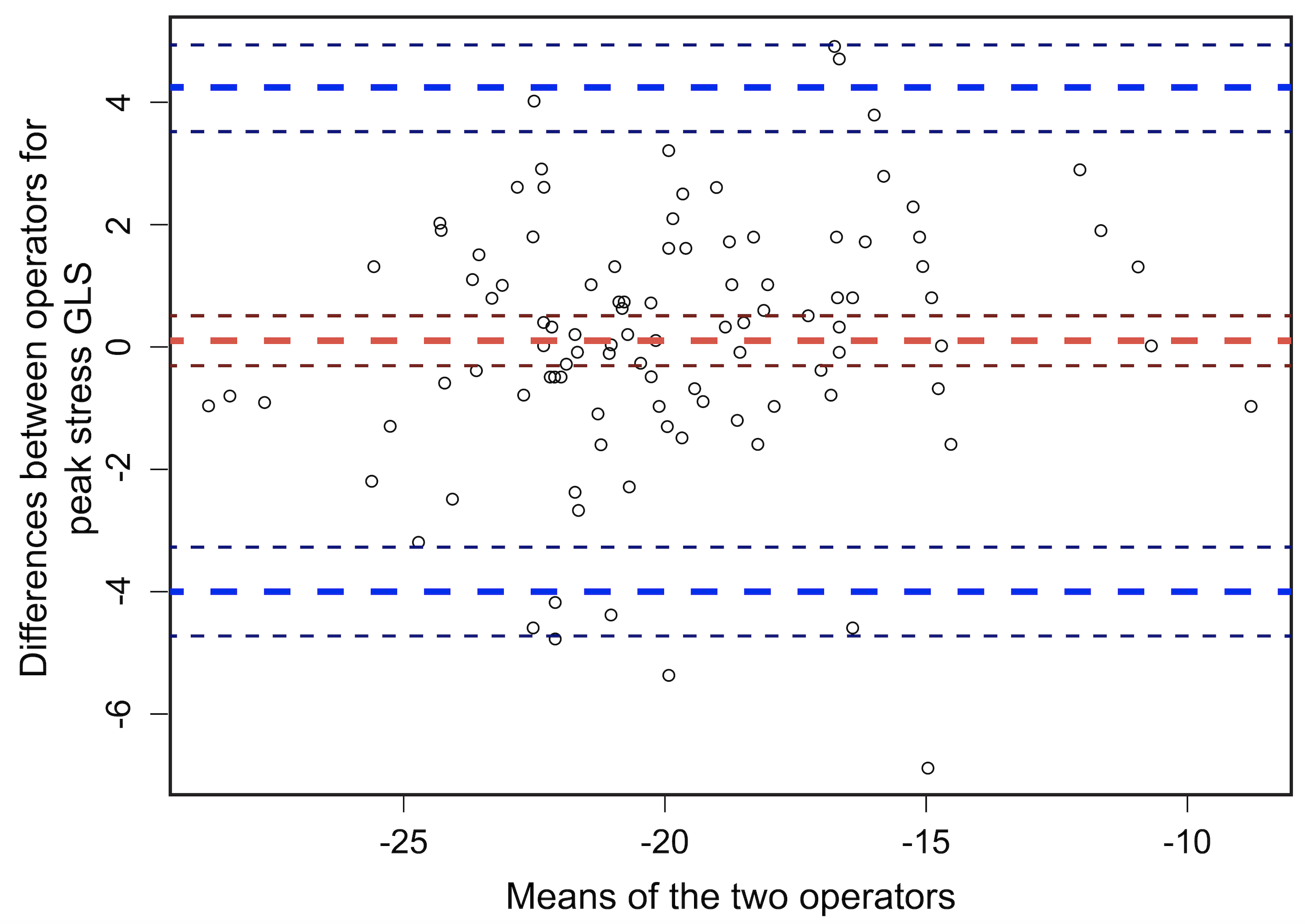
**

**Figure S3.** Bland-Altmann plot for strain at baseline of the apical segments (segments 13-17)

**
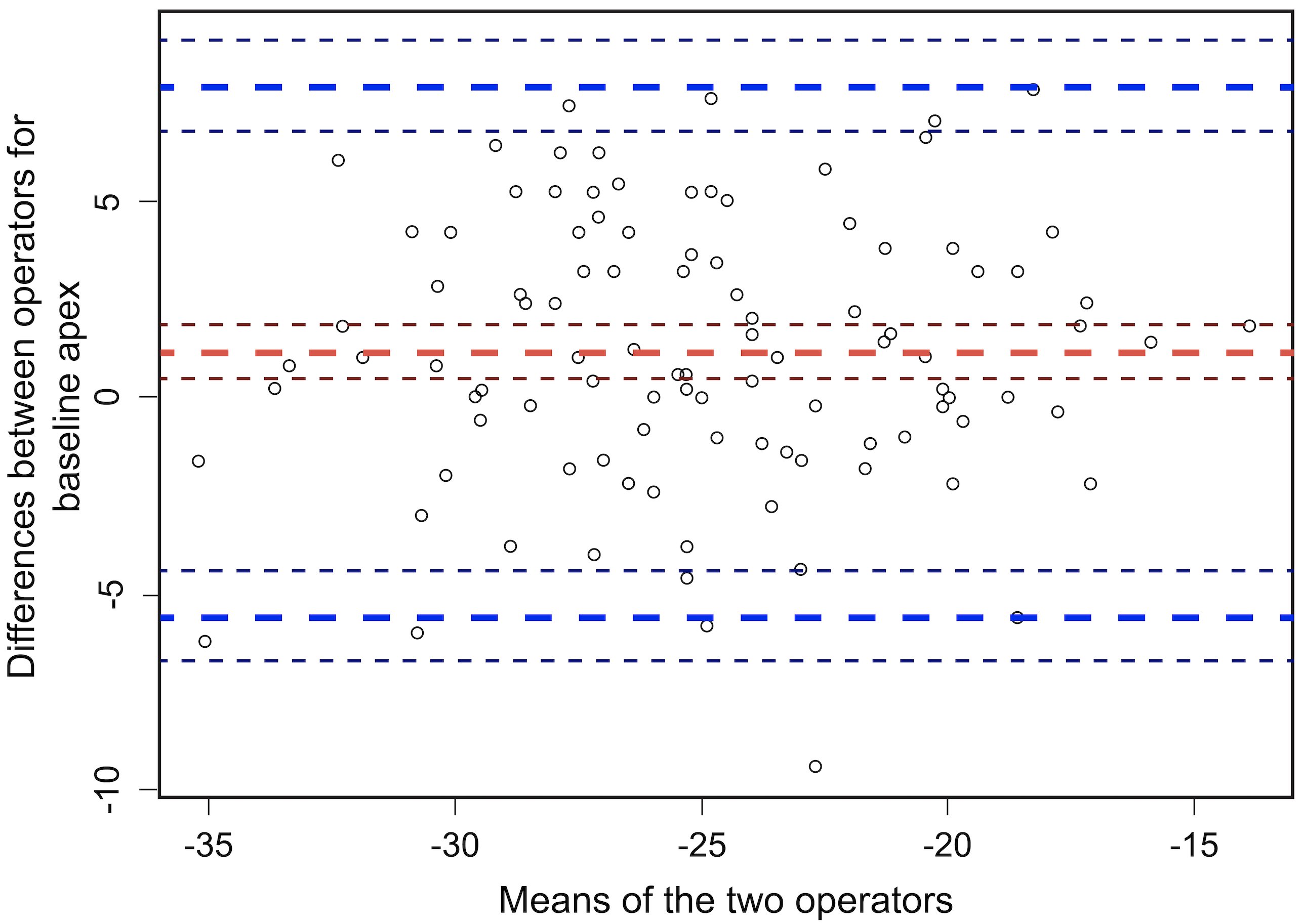
**

**Figure S4.** Bland-Altmann plot for strain at peak stress of the apical segments (segments 13-17)

**
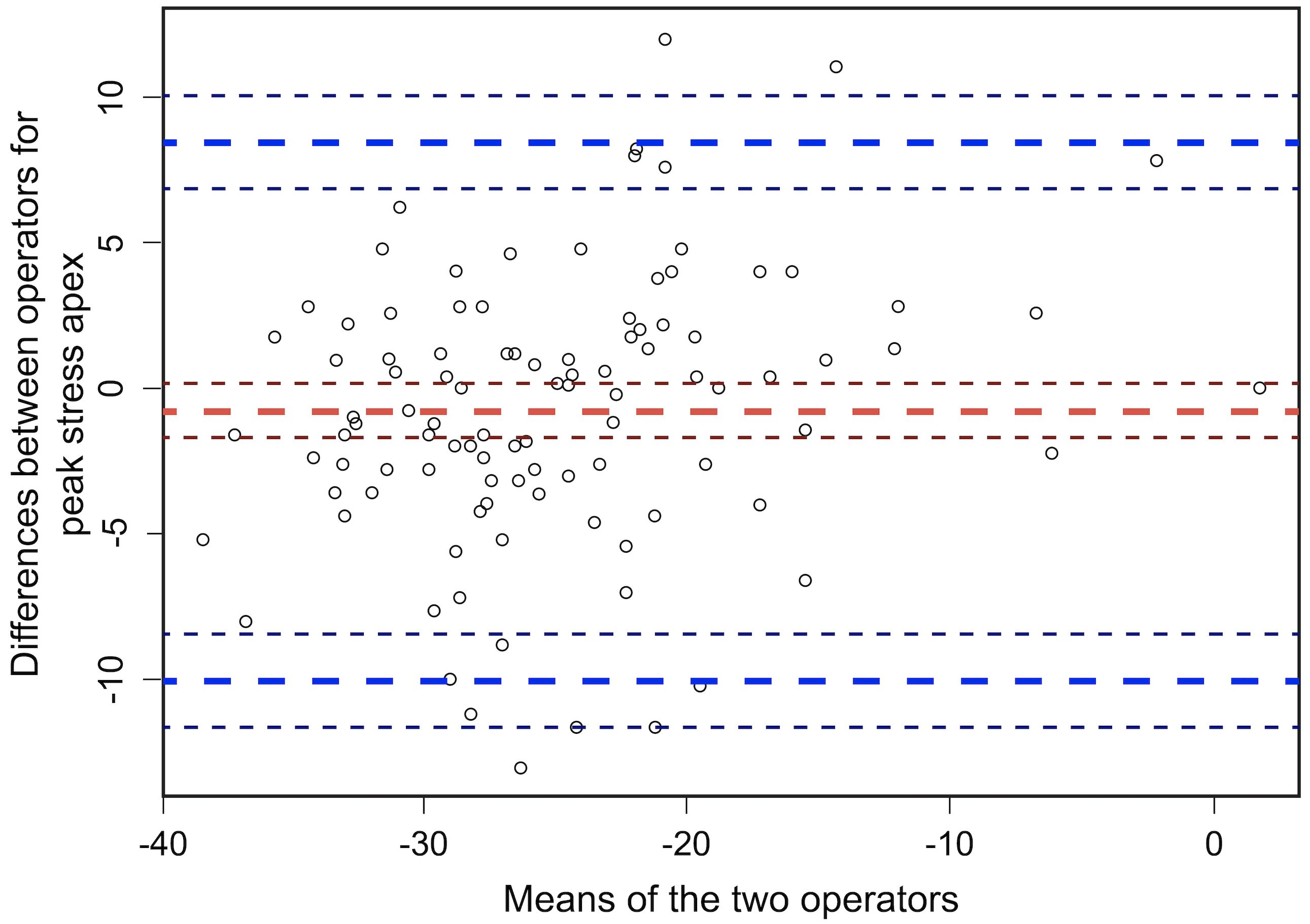
**
